# Teff-Based Probiotic Functional Beverage Fermented with *Lactobacillus rhamnosus* and *Lactobacillus plantarum*

**DOI:** 10.3390/foods10102333

**Published:** 2021-09-30

**Authors:** Sendeku Takele Alemneh, Shimelis Admassu Emire, Bernd Hitzmann

**Affiliations:** 1 Food Engineering, Addis Ababa Institute of Technology, Addis Ababa University, King George VI St, Addis Ababa 1000, Ethiopia; shimelis.admassu@aait.edu.et; 2 Process Analytics and Cereal Science, Institute of Food Science and Biotechnology, University of Hohenheim, 70559 Stuttgart, Germany; bernd.hitzmann@uni-hohenheim.de

**Keywords:** food fermentation, functional food, *Lactobacillus plantarum*, *Lactobacillus rhamnosus*, probiotic food, teff-based substrate

## Abstract

Consumers are demanding healthier foods, and the increasing drawbacks associated with dairy-based products have driven efforts to find plant-based probiotic alternatives. Consequently, this study aimed to evaluate the suitability of a teff-based substrate for delivering the potential probiotics, *Lactobacillus rhamnosus* GG (LGG) and *Lactobacillus plantarum* A6 (LA6) with a view to developing probiotic functional beverages. Single-strain and mixed-strain fermentations were performed without any pH control. In single-strain fermentation, LA6 grew to 8.157–8.349 log cfu/mL. Titratable acidity (TA) and pH were measured between 0.513–1.360 g/L and 4.25–3.91, respectively. The explored optimum variables were fermentation time (15 h) and inoculum (6 log cfu/mL). As a result of fermentation, maltose and glucose decreased, but lactic and acetic acids increased. In mixed-strain fermentation, LGG and LA6 were able to grow to 8.247 and 8.416 log cfu/mL, respectively. The pH, TA, lactic, and acetic acids varied between 6.31–3.92, 0.329–1.501 g/L, 0–1672 mg/L, and 20–231.5 mg/L, respectively. In both fermentations, microbial growth reached the stationary phase close to a pH of 4.21–4.82 while sugars were not consumed completely. Less than 5% ethanol was detected, which indicated a non-alcoholic beverage. A combination of the two evaluated lactobacilli strains reduced fermentation time. In conclusion, a substrate made of whole grain teff flour without any supplement could be used as a substrate to produce functional probiotic beverages.

## 1. Introduction

Increasing consumer awareness of the direct relationship between diet and health has opened up a huge market demand for new functional foods with health benefits [1]. The search by vegetarian consumers for health-promoting foods makes them an attractive commercial target group for the food industry [2]. Regular consumption of health-promoting foods could help consumers to take care of themselves and, at the same time, reduce their risk of disease [3]. Functional foods contain a range of ingredients that offer health benefits beyond basic nutrition [4]. Probiotic-containing foods rank among them. They contain a minimum typical level of 6–7 log cfu/mL or g of probiotic strains in food. This level of viable microbial count at the time of consumption can bring about beneficial modifications to the host’s intestinal microbiota [5]. All in all, probiotic foods are becoming more interesting, and societal acceptance of these food products is on the rise [6]. According to the definition of the FAO/WHO 2002, probiotics are beneficial microorganisms that deliver health benefits when administered in sufficient quantities [7].

For a very long time now, dairy products have been used as a medium of probiotic delivery [8]. However, there are disadvantages, i.e., lactose intolerance and cholesterol coupled with a shift towards the use of non-dairy probiotic foods inspired by vegetarianism. Cereals could, therefore, constitute an alternative way of catering for the food preferences and demands of people who have concerns about dairy products. Cereals are important sources of protein, carbohydrates, vitamins, minerals, and fiber for the global population [9]. Whole grain cereals, in particular, are important for probiotics’ growth, and their nondigested carbohydrates act as prebiotics. Moreover, cereals have functioned as encapsulation materials to boost probiotic stability [10]. Against this backdrop, cereals have been found to be suitable probiotic carriers [8], and have been used in the design of probiotic functional foods [11].

Teff is an annual crop in the Poaceae (grass) family, and it has a significant role in the food security. It is an indigenous food crop in Ethiopia and Eritrea for the production of a variety of traditional foods such as injera, kitta and tella [12]. Teff contains gluten-free protein and has high levels of essential amino acids and minerals, a low glycemic index, a high content of crude fiber, and a longer shelf life. As a result, the global demand for teff is increasing [13]. Furthermore, teff constitutes an alternative for consumers with a lifelong intolerance to gluten [14]. However, no information is available on the suitability of a teff-made substrate for the growth of probiotic lactic acid bacteria.

This research aimed to evaluate the suitability of a teff-based substrate for the growth of the probiotics, LGG and LA6, to produce probiotic functional beverages. The single-strain fermentation of a teff-based substrate (formulated without the addition of a supplement) was investigated with a view to analyzing substrate and inoculum formulations.

## 2. Materials and Methods

### 2.1. Materials

Whole teff (*Eragrostis tef*) grain (30 kg) was sourced from Debrezeit Agricultural Research Center, Debrezeit, Ethiopia in December 2019. Its variety name is DZ-Cr-438. The grain was milled using an attrition disc mill, which is commonly used for commercial teff grinding in Ethiopia. The other whole teff grain flour (25 kg) was purchased from Teff-shop de, Manuel Boesel, Homburger Str.49a, 61191 Rosbach von der Höhe, Germany. Freeze-dried strains of LA6 (LMG 18053, BCCM, Gent, Belgium) and LGG (LMG 18243, BCCM, Gent, Belgium) were provided via the Department of Process Analytics and Cereal Science, Institute of Food Science and Biotechnology, University of Hohenheim, 70559 Stuttgart, Baden-Württemberg, Germany.

### 2.2. Starter Culture, Storage and Substrate Preparation

Freeze-dried strains of LA6 (LMG 18053, BCCM, Gent, Belgium) and LGG (LMG 18243, BCCM, Gent, Belgium) were recovered and maintained at 6 °C until utilization. The LA6 starter culture was prepared using the same condition [15], which was obtained by overnight incubation in a refrigerated incubator (BINDER GmbH, KB 115, Tuttlingen, Germany) at 30 °C in sterile MRS (DE MAN, ROGOSA and SHARPE) broth whereas the LGG starter culture was prepared as described by [16]. The LGG inoculum of LGG was obtained by overnight incubation at 37 °C in sterile MRS broth. For inoculation, starter cultures were harvested by centrifugation (Mega star 600R, Leuven, Belgium) at 3000× *g*, 4 °C for 15 min. The pellets of cells were washed in a sterile saline solution (0.9% NaCl) and then centrifuged again using the same condition. The supernatant was removed and the pellets were re-suspended in a saline solution to form a cell suspension of approximately 9 log cfu/mL. This constituted the inoculum and was kept at 6 °C.

The microbial strains were stored deep-frozen at −70 °C in 40% glycerol [17]. The glycerol stock was prepared by mixing 40% glycerol with 60% MRS broth and then autoclaved. It was then transferred to 1.5 mL sterile screw cap tubes with an overnight culture of LA6 and LGG. In the next phase, it was vortexed, labelled, and stored at −70 °C. The teff-based substrates were prepared by mixing whole grain teff flour with distilled water in 4%, 5.5%, and 7% *w*/*v*. Each substrate was heated in a water bath (GFL-1083, Burgwedel, Germany) for 15 min at 85 °C and then sterilized (SHP Laboklav, 160-MSLV, Satuelle, Germany). Prior to inoculation, the substrate was left to cool in a Hera Safe safety cabinet (Kendro Laboratory Products GmbH, KS 12, Hanau, Germany).

### 2.3. Enumeration of the Viable Microbial Count

The LA6 viability was quantified via plated MRS agar in line with the Miles and Mishra method [16]. The MRS agar was prepared by adding 15 g agar to 1 liter of MRS broth. The suspensions of fermented beverage were sampled every 3 h including initially, and serial 10-fold dilutions were prepared in a saline solution. Using a calibrated 10–100-μL micropipette, 50-μL drops of an appropriate dilution were placed on MRS agar plates, and incubated at 30 °C for 24 h [18]. The initial viable density of LGG was achieved using the above-mentioned condition, but it was incubated for 48 h. In addition, anaerobiosis and aerobiosis conditions with different temperatures (30 and 37 °C) and incubation time (24, 36, and 48 h) were tested for the selective enumeration of LA6 and LGG. Finally, plates containing 30–300 colonies were enumerated and recorded as LA6 and LGG colony-forming units (cfu).

### 2.4. Substrate Fermentation with a Single Strain

The overnight cultures of LA6 with inoculums of 5, 6, and 7 log cfu/mL were inoculated to substrates. Fermentation was performed for 24 h without any pH control in a 1-L Erlenmeyer flask containing 0.5 L of working volume, and incubated in an IKA Shaker (IKA, KS 4000 i, Staufen, Germany) for 24 h at 30 °C with 150 rpm. Samples were taken aseptically every 3 h to analyze viable microbial count, pH, and TA in terms of lactic acid.

### 2.5. Experimental Design

A completely randomized design for two factors with three level combinations was used. The experiment results obtained for the fermentation of substrates inoculated with LA6 are presented in Table 1.

Substrate, teff flour suspension in distilled water; Inoculum, *Lactobacillus plantarum* A6 initial density.

### 2.6. Fermentation of Substrate with Mixed Strains

Mixed-strain (LA6 and LGG) fermentation was performed for 15 h with inoculation of 6 log cfu/mL. The inoculation ratio was 1:1, and fermentation was performed without any pH control in 1-L Erlenmeyer flasks containing 0.5 L of fermenting media. The fermentation media were incubated in an IKA Shaker (IKA, KS 4000 i, Staufen, Germany) at 37 °C with 150 rpm. The samples for the analyses of viable microbial count, pH, TA, sugar, and organic acid were taken aseptically at 3 h intervals during fermentation.

### 2.7. Analytical Methods

#### 2.7.1. The pH and TA

The analyses of pH and TA were determined using the American Association of Cereal Chemists’ methods 02–31.01 and 02–52.01, respectively [19]. The pH of the samples was measured with the pH electrode SJ 114 using a digital pH meter (Xylem, pH730, GS1 Sweden), which was calibrated at pH 4.0 and 7.0. The TA was determined by neutralizing the acid present in the sample using 0.1 N NaOH with the addition of phenolphthalein as 1% in alcohol [20]. For pH and TA analysis, samples were centrifuged at 3000× *g*, 4 °C for 15 min and the supernatant was stored in a deep-frozen state at −20 °C.

#### 2.7.2. Organic Acid and Sugar

The changes in organic acid and sugar were analyzed in samples using HPLC every 3 h. The samples were centrifuged at 3000× *g*, 4 °C for 15 min, and the supernatant was filtered with a 0.45-μm polypropylene membrane (VWR, Darmstadt, Germany). After centrifugation and filtration, the sample supernatant was examined by HPLC (ProStar, Variant, Walnut Creek, CA, USA) equipped with refractive index detector to determine maltose, glucose, lactic acid, acetic acid, and ethanol. Then 20 μL of the sample were injected into a Rezex ROA-organic acid H^+^ (8%) column (Phenomenex, Aschaffenburg, Germany) operated at 70 °C with 5 mM H_2_SO_4_ as an eluent with a 0.6-mL/min flow rate. The analytes were calculated in Software Galaxie^TM^ Chromatography (Varian, Walnut Creek, CA, USA).

### 2.8. Statistical Analysis

The kinetic parameters of microbial growth, pH, and TA were determined with the DMFit tool, which is a mechanistic model [21]. For curve plots, Graphpad Prism 9 (Graphpad Software LLC, San Diego, California, USA,) was used. One-way analysis of variance using the Tukey multiple comparison method was used to observe significant (*p* < 0.05) differences using Minitab 18.1 software (2017Minitab, Inc., State College, PA, USA). The results of means ± standard deviation are reported.

## 3. Results and Discussion

### 3.1. Growth of Lactobacillus plantarum A6 (LA6) in Single-Strain Fermentation

**Figure 1:**
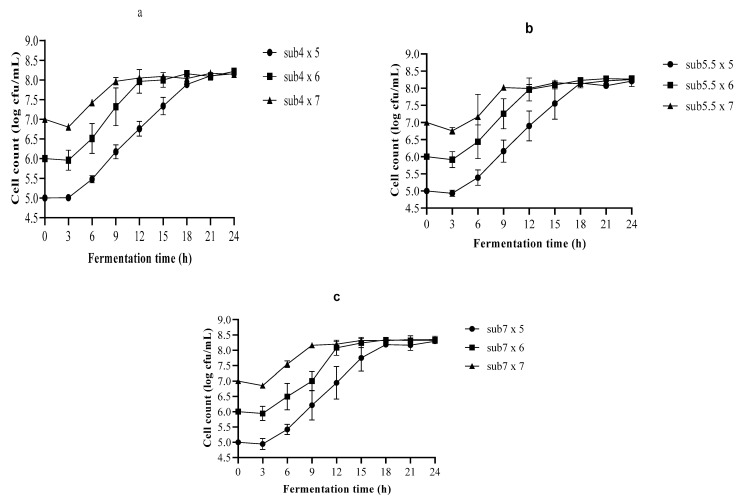
Growth of *Lactobacillus plantarum* A6 inoculated in (**a**) 4% *w*/*v*, (**b**) 5.5% *w*/*v*, and (**c**) 7% *w*/*v* substrate; sub4 × 5, 4% *w*/*v* substrate inoculated with 5 log cfu/mL; sub4 × 6, 4% *w*/*v* substrate inoculated with 6 log cfu/mL; sub4 × 7, 4% *w*/*v* substrate inoculated with 7 log cfu/mL; sub5.5 × 5, 5.5% *w*/*v* substrate inoculated with 5 log cfu/mL; sub5.5 × 6, 5.5% *w*/*v* substrate inoculated with 6 log cfu/mL; sub5.5 × 7, 5.5% *w*/*v* substrate inoculated with 7 log cfu/mL; sub7 × 5, 7% *w*/*v* substrate inoculated with 5 log cfu/mL; sub7 × 6, 7% *w*/*v* substrate inoculated with 6 log cfu/mL; sub7 × 7, 7% *w*/*v* substrate inoculated with 7 log cfu/mL.

In this study, slurry made of whole-grain teff flour (hereafter, substrate) was used. The substrate was inoculated with a culture suspension of LA6 to achieve cell growth of 6–7 log cfu/mL. This is the minimum required number at time of consumption based on 0.1-L daily intake [22]. Probiotic cultures need a long fermentation time to reach a low pH. However, the food industry always prefers a short fermentation time to increase plant output and reduce microbial contamination [16]. As a solution to this challenge, this research used amylolytic LA6 bacteria integrated with substrate pre-heating. The growth of LA6 observed in 24-h fermentation is presented in Figure 1.

According to Figure 1, all substrates were suitable for LA6 growth. At the beginning of the 24-h fermentation of 4% *w*/*v* substrate, the initial cell densities were 5, 6, and 7 log cfu/mL. They grew, respectively, to 8.157, 8.218, and 8.146 log cfu/mL. However, significant (*p* < 0.05) growth change was not observed for the first 6 h. Exponential growth phases were observed up to 12 h and 18 h of fermentation with inoculums of 6 and 5 log cfu/mL, respectively. With the same substrate fermentation, microbial growth decreased from 7 to 6.799 log cfu/mL during the first 3 h. This might be due to the environmental adaptation of cells and competition within microbial strains to use the available sugars. Afterwards, there was a significant (*p* < 0.05) increase in cell growth to 12 h, and it subsequently reached the stationary phase.

Additionally, LA6 grew in 5.5% *w*/*v* substrate from 5, 6, and 7 log cfu/mL to 8.206, 8.268, and 8.253 log cfu/mL, respectively. In these fermentations, there was no significant (*p* < 0.05) growth change in the first 6 h, except for the 5 log cfu/mL inoculum. It showed a slight growth difference at 6 h. It then grew exponentially up to 15, 12, and 9 h with inoculums of 5, 6, and 7 log cfu/mL, respectively. Furthermore, in the 24-h fermentation of 7% *w*/*v* substrate, LA6 grew initially from 5, 6, and 7 log cfu/mL to 8.294, 8.323, and 8.349 log cfu/mL, respectively. In this substrate, major growth change was not observed for the first 6 h. Nonetheless, the inoculum of 7 log cfu/mL showed a significant (*p* < 0.05) increase in cells at 6 h. Subsequently, the rapid growth of cells continued up to 15, 12, and 9-h, respectively with inoculums of 5, 6, and 7 log cfu/mL.

In all substrate fermentations, the growth of LA6 with inoculums of 5, 6, and 7 log cfu/mL increased rapidly during the exponential phase and reached the stationary phase at approximately 15–18, 12, and 9–12 h, respectively. Overall, the final growth of LA6 varied between 8.146–8.218 log cfu/mL without any decreasing phase. Although not significant, slight differences in cell counts were observed in substrate concentrations. This might be due to buffering capacity of substrates. Buffering capacity might be increased with concentration of substrates since a greater amount of hydrogen ions might be presented in fermenting medium at higher substrates concentration. According to this study, inoculums have no major effect on the final growth of LA6. Moreover, substrates made from whole teff flour in distilled water were suitable for the growth of LA6. It remained viable at counts beyond 6–7 log cfu/mL [23], which is the minimum required number at time of consumption to achieve a health-promoting effect.

The growth of LA6 obtained in this study was higher than for 7–8 log cfu/mL, which was found during the 24-h fermentation of a rice-based substrate [24]. The bacterial population of LA6 detected in the 18-h fermentation of pearl millet slurry and MRS-starch changed by between 6.982–8.820 log cfu/mL and 7.820–8.176 log cfu/mL, respectively [25]. Similar growth of *Lb. plantarum* (8.17 log cfu/mL) was reported in the 30-h fermentation of an oat bran-based substrate [26]. Moreover, comparable cell counts of *Lb. plantarum* in the 24-h fermentation of malt, barley, and a mix of malt and barley were indicated as 8.59, 7.91, and 8.53 log cfu/mL, respectively [22].

Furthermore, the growth of LA6 correlated well with counts of *Lb. plantarum* (7–8.2 and 7.9 log cfu/mL), which were investigated in the 36-h fermentation of oats and barely [27]. Moreover, the similar growth of *Lb. acidophilus*, *Lb. plantarum*, and *Lb. reuteri* (7.76–8.20 log cfu/mL) was reported in the 10-h fermentation of starch-free extracts of oats, barely, and malt [28]. According to LUF Emma, GTS Melissa, T Tiina, J Mari, GP-N Maria, K Hannu and P-F Carme [29], a higher inoculum (8 log cfu/mL) and final growth (9.5 log cfu/mL) of *Lb. plantarum* were reported in the 6-h fermentation of 15% *w*/*v* quinoa-based substrate. A similar count of *Lb. plantarum* (8.34 log cfu/mL) was observed in the 24-h fermentation of mature coconut inoculated with 7 log cfu/mL [30].

The growth rate and lag phase of LA6 in all beverage formulations varied between 0.215–0.313 h^−1^ and 3.490–5.173 h, respectively (Table 2). The lowest growth rate and lag phase were observed in the 4% *w*/*v* substrate inoculated with 5 log cfu/mL. The highest growth rate and lag phase were found in the fermentation of 5.5% *w*/*v* substrate using 6 log cfu/mL inoculum. Almost the same result was obtained in the fermentation of 7% *w*/*v* substrate inoculated with 6 log cfu/mL. Similar lag values (3.37 and 4.38 h) were noted in an oat substrate fermentation inoculated with *Lb. acidophilus* and *Lb. casei*, respectively [31]. The DMFit, a mechanistic dynamic model, estimated kinetic growth parameters with R-squared above 95%, and standard error of fitting below 1%. This might be an indication that kinetic parameters were estimated with a low degree of uncertainty.

### 3.2. The pH and TA in Single-Strain Fermentation

**Figure 2:**
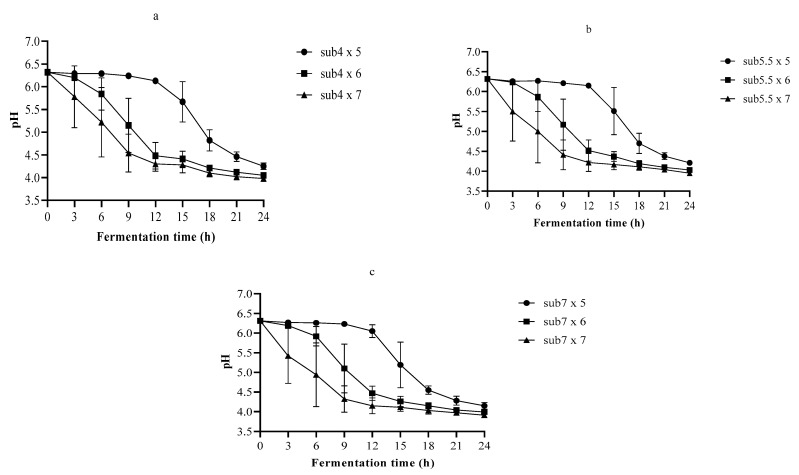
The pH changes during fermentation of (**a**) 4% *w*/*v*, (**b**) 5.5% *w*/*v*, and (**c**) 7% *w*/*v* substrate with *Lactobacillus plantarum* A6; sub4 × 5, 4% *w*/*v* substrate inoculated with 5 log cfu/mL; sub4 × 6, 4% *w*/*v* substrate inoculated with 6 log cfu/mL; sub4 × 7, 4% *w*/*v* substrate inoculated with 7 log cfu/mL; sub5.5 × 5, 5.5% *w*/*v* substrate inoculated with 5 log cfu/mL; sub5.5 × 6, 5.5% *w*/*v* substrate inoculated with 6 log cfu/mL; sub5.5 × 7, 5.5% *w*/*v* substrate inoculated with 7 log cfu/mL; sub7 × 5, 7% *w*/*v* substrate inoculated with 5 log cfu/mL; sub7 × 6, 7% *w*/*v* substrate inoculated with 6 log cfu/mL; sub7 × 7, 7% *w*/*v* substrate inoculated with 7 log cfu/mL.

**Figure 3:**
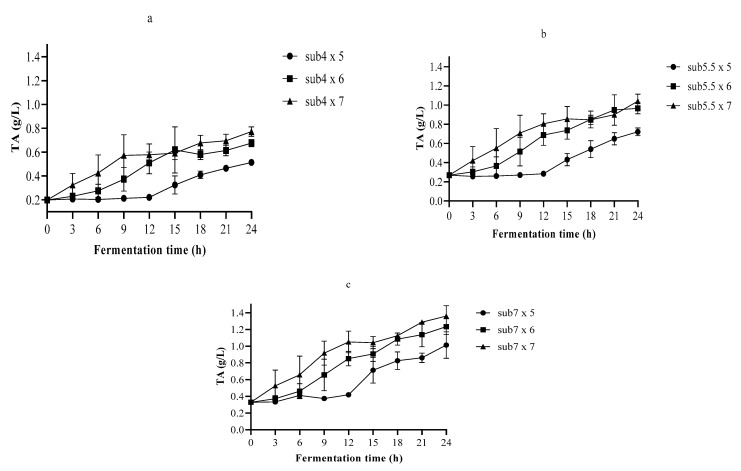
Titratable acidity changes during fermentation of (**a**) 4% *w*/*v*, (**b**) 5.5% *w*/*v*, and (**c**) 7% *w*/*v* substrate with *Lactobacillus plantarum* A6; sub4 × 5, 4% *w*/*v* substrate inoculated with 5 log cfu/mL; sub4 × 6, 4% *w*/*v* substrate inoculated with 6 log cfu/mL; sub4 × 7, 4% *w*/*v* substrate inoculated with 7 log cfu/mL; sub5.5 × 5, 5.5% *w*/*v* substrate inoculated with 5 log cfu/mL; sub5.5 × 6, 5.5% *w*/*v* substrate inoculated with 6 log cfu/mL; sub5.5 × 7, 5.5% *w*/*v* substrate inoculated with 7 log cfu/mL; sub7 × 5, 7% *w*/*v* substrate inoculated with 5 log cfu/mL; sub7 × 6, 7% *w*/*v* substrate inoculated with 6 log cfu/mL; sub7 × 7, 7% *w*/*v* substrate inoculated with 7 log cfu/mL.

The TA is a better predictor of the effect of organic acid on the flavor of a particular food. However, the microbes’ ability to grow depends on the pH rather than on the TA [32]. In all 24 h fermentations, the lowest pH and the highest TA recorded were 3.91 and 1.36 g/L, respectively (Figure 2 and Figure 3).

The organic acids produced during the fermentation process lowered the pH and increased the TA of the fermenting medium because fermentation was performed without any pH control. The pH fell from 6.26 to 4.30–4.82 towards the end of the exponential growth of LA6 in 4% *w*/*v* substrate fermentation, which was inoculated with 5, 6, and 7 log cfu/mL (Figure 2). Furthermore, in the case of the 5.5% *w*/*v* substrate fermented with the above-mentioned inoculums in a similar growth phase, the pH dropped from 6.25 to 4.41–5.51. Moreover, at the end of the exponential growth phase in 7% *w*/*v* substrate fermentation, the pH diminished from 6.28 to 4.32–5.19. Overall, at the end of the 24-h fermentation, the pH fluctuated between 4.25–3.91 depending on the beverage formulations. A pH of 4–4.5 is vital for the survival of the fermented beverage during storage [29]. Additionally, many pathogens in fermented foods cannot grow at a pH ≤ 4.0 [33].

Figure 3 shows that the TA values did not increase significantly (*p* < 0.05) in all the first 9-h fermentations, except in the 7% *w*/*v* substrate inoculated with 6 log cfu/mL. The substrate fermented with the inoculum of 7 log cfu/mL showed that TA started increasing meaningfully (*p* < 0.05) at 6 h. Throughout the 24-h fermentation, the maximum TA changed between 0.513–1.36 g/L. The highest TA was obtained in the 7% *w*/*v* substrate inoculated with 7 log cfu/mL, which has been deemed to have better sensory quality [33]. The smallest TA was observed in the 4% *w*/*v* substrate fermented with the inoculum of 5 log cfu/mL. In general, throughout fermentation, a drop in pH and an increase in TA were observed. This demonstrated the action of LA6 during fermentation involving the production of organic acids.

In the 24-h fermentation of the single strain, the pH drop rate and the lag phase changed between 0.166–0.267 h^−1^ and 0–10.622 h, respectively (Table 3). The increase in the TA rate and the lag phase varied between 0.021–0.046 h^−1^ and 0–10.685 h, respectively. The highest pH drop rate and TA increase were 0.267 and 0.046 h^−1^, respectively. The pH drop and the TA increase did not present a lag phase in all fermentations inoculated with 7 log cfu/mL. Likewise, there was no TA lag phase in 5.5 and 7% *w*/*v* substrate fermentation inoculated with 6 log cfu/mL. The DMFit estimated the kinetic parameters of pH and TA using decent R-squared and standard error of fitting (Table 3).

### 3.3. Optimization of the Fermentation Process Using Nelder–Mead Simplex

Regardless of the substrate concentration in all the fermentations, the growth of LA6 reached stationary phase when viable counting reached approximately 8 log cfu/mL (Figure 1). In addition, at this point the pH was around 4.5. This demonstrated that the substrate concentrations were not the controlling growth factor of LA6. For that reason, the pH might be the limiting growth factor. Although final cell populations were nearly the same, the stationary phase was achieved at different times. For instance, for all substrate fermentation inoculated with 5 log cfu/mL, the stationary phase was reached between approximately 15–18 h. In addition, the inoculums of 6 and 7 log cfu/mL reached the stationary phase at around 12 and 9 h, respectively.

Afterwards, optimization was undertaken using the influencing variables time (9 to 15 h) and inoculum (5–7 log cfu/mL). Concerning the substrate concentrations, the one with almost 7% *w*/*v* was preferred because it had the highest TA (Figure 3), which might be important for sensory quality. The results of the Nelder–Mead simplex method are given in Table 4. The optimization objective was to maximize the Quality Function (QF) based on microbial growth and pH.

(1) $$\mathrm{QF}=\left(\frac{\mathrm{Cell}\;\mathrm{count}\;\mathrm{in}\log\frac{\mathrm{cfu}}{\mathrm{mL}}}{8}\right)+\left(\frac{4}{\mathrm{pH}}\right)$$

Here, denominator 8 and numerator 4 were chosen because the growth of LA6 reached the stationary phase when cell counts were recorded of approximately 8 log cfu/mL with a pH of about 4.

The growth of LA6, pH, and TA including the quality function did not show a significant (*p* < 0.05) difference from the third experiment on (Table 4). However, the highest cell count of LA6, and the production of lactic and acetic acids were observed in in the sixth experiment. The sixth experiment was, therefore, used as the optimum point where the influencing variables were 15 h and the 6 log cfu/mL. Overall, the cell counts varied between 7.781–8.400 log cfu/mL, which is above 6 log cfu/mL. To generate health benefits, the minimum probiotics’ number should be 6–7 log cfu/mL or g of food at time of consumption [34]. This helps to limit washout and to guarantee that the benefits of probiotics will be increased in a sustained manner [35].

During the production of lactic and acetic acids, the glucose medium was eventually consumed completely. Similarly, maltose decreased as fermentation time and inoculum increased while lactic and acetic acids increased over time (Table 5). The glucose was completely consumed in the 15-h, 14.25-h, and 15-h fermentations. They were inoculated with 6.226, 6.068, and 6 log cfu/mL, respectively. However, maltose production was detected at points where glucose was consumed completely, which showed that LA6 preferably utilized glucose as a carbon source rather than maltose. The manufacture of lactic and acetic acids is directly related. However, ethanol showed an opposite trend, i.e., as acids increased, ethanol decreased. This might be due to the ability of LA6 to oxidize ethanol to acetic acid under aerobic conditions. According to the previous study by [36], together lactic and acetic acids reduce ethanol production via the fermentation of corn mash with *Saccharomyces cerevisiae*. These inhibitory effects are more apparent at higher temperatures.

### 3.4. Selective Enumeration of Tested Lactobacilli Strains

Anaerobiosis and aerobiosis conditions with different temperatures and incubation time were examined for the selective enumeration of *Lactobacillus rhamnosus* GG (LGG) and LA6 (Table 6). Aerobiosis incubation for 48 h at 30 °C was selected for the counting of LA6 and LGG in the same sample. Samples with mixed strains of LA6 and LGG were incubated on MRS agar plates for 48 h. Viable counts of LA6 were obtained at approximately 24 h of incubation, but LGG did not grow at this time. However, afterwards, both microbes grew at about 48 h of incubation, and total viable counts were then recorded. Finally, the difference was deemed to constitute viable counts of LGG.

### 3.5. Growth of LA6 and LGG, pH, and TA in Mixed-Strain Fermentation

**Figure 4:**
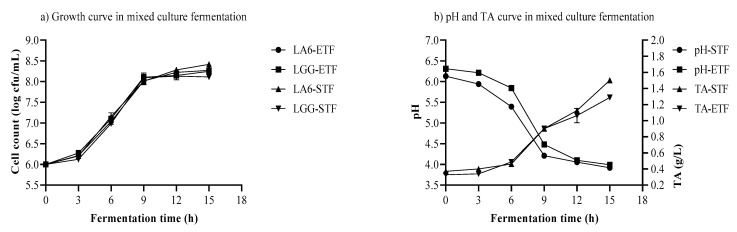
(**a**) Growth of *Lactobacillus plantarum* A6 and *Lactobacillus rhamnosus* GG, and (**b**) changes in pH & TA, (titratable acidity) in mixed culture fermentation; LA6-ETF & LGG-ETF, teff flour samples grown in Ethiopia inoculated with *Lactobacillus plantarum* A6 & *Lactobacillus rhamnosus* GG; LA6-STF & LGG-STF, teff flour grown in South Africa inoculated with *Lactobacillus plantarum* A6 & *Lactobacillus rhamnosus* GG; pH-ETF & TA-ETF, pH and TA found in teff flour samples grown in Ethiopia; pH-STF & TA-STF, pH and TA found in teff flour samples grown in South Africa.

Mixed-strain fermentations were performed with optimum variables (Table 4). Mixed strains of LA6 and LGG were used for the individual fermentation of ETF and STF substrates. The results of the viable microbial counts, TA, and pH during mixed-strain fermentation are presented in Figure 4. The probiotic strains of LA6 and LGG were able to grow from 6 to 8.269 and 8.416 log cfu/mL, respectively. Similar growth of LGG was reported in the fermentation of barley and rye flour [37]. Conversely, the lower count of LGG (6.73 log cfu/mL) was investigated in mixed pineapple and jussara substrates fermented for 72 h at 37 °C [38].

In mixed-strain 15-h fermentation, the pH fell close to 3.99 and 3.92, respectively, in the substrates of ETF and STF. This revealed that the 6 log cfu/mL inoculum was able to lower the pH. Food formulations with pH values around 3.5–4.5 are necessary as they contribute to a decreased pH in the gastrointestinal tract, and enhance the stability of consumed probiotics [39]. Under similar conditions, the highest TA of 1.288 and 1.501 g/L was found in substrates of ETF and STF, respectively. These differences in pH and TA could be attributable to individual available sugars found in each substrate. The conversion of accessible sugars to organic acids by LA6 and LGG led to a drop in pH and an increase in TA. A low pH of around 4.0 in the beverage is vital for the safety, taste, and aroma of the beverage [1]. A decreasing pH over the course of fermentation would constitute a harsh environment for certain spoilage bacteria [40].

In this study, some differences were observed in the cell counts, pH, and TA between the single-strain and mixed-strain fermentations of the same substrate and inoculum. This might be due to interactions between strains. Comparable maximum growth of LA6 was found in single-strain and mixed-strain fermentations. However, the same cell counts were achieved over a short period of time with decreasing pH during mixed-strain fermentation. This was not observed in single-strain fermentation.

While this does not constitute a major difference, a lower growth rate was observed for LA6 than for LGG in a similar ETF substrate, but the lag phase showed the reverse trend (Table 7). Similarly, the growth rate of LA6 was lower compared to LGG in the same STF substrate. In addition, the same comparison was observed in the lag phase. A higher pH drop rate was exhibited in the ETF substrate. However, a greater increase in the TA rate was found in the STF substrate. The lag phase of TA was not achieved in the ETF substrate. The longer pH lag phase was observed with LGG inoculations of 7.90, 8.70, and 6.50 h, respectively in amaranth grain, buckwheat flour, and millet grain. In these substrates, the respective pH drop rates were calculated as 0.355, 1.063, and 0.244 h^−1^ [37].

Overall, a slight difference in fermentation parameters was observed between the ETF and STF substrates which could be attributable to the available sugars. Using a similar formulation, a lower growth rate and a higher lag phase of LA6 were investigated in single-strain compared to mixed-strain fermentation. Moreover, the decreasing pH rate and the increasing TA rate were higher in mixed-strain fermentation.

### 3.6. Organic Acid and Sugar in Mixed-Strain Fermentation

Lactic acid, acetic acid, maltose, glucose, and ethanol derived from substrates of ETF and STF are presented in Table 8 and Table 9. Examination of glucose and maltose revealed a significant (*p* < 0.05) decrease. However, the highest values were detected at 6-h fermentation (Table 8). From 12-h fermentation, glucose was consumed entirely, but maltose remained. This was an indication that the evaluated lactobacilli strains preferred to consume glucose rather than maltose (a similar situation occurred during single-strain fermentation). The lactic and acetic acids increased throughout fermentation and continued thereafter whereas cell growth remained at a stationary phase. In addition, the ETF substrate produced ethanol during 9–12-h fermentation and afterwards it returned to zero. The metabolites produced correlated with glucose and maltose i.e., at higher glucose and maltose levels, lower metabolites were found and vice-versa.

Glucose was progressively consumed in the medium while lactic and acetic acids were increasing. However, lactic acid fell at 12-h fermentation (Table 9). This variation of lactic acid could be associated with the fluctuation of sugar which, in turn, might be due to the further breakdown of starch to mono/disaccharides in the substrates used owing to amylase activity within lactic acid bacteria. Glucose was completely consumed at 15 h, but maltose was still present. The common enzyme detected in LA6 was alpha-amylase, which can hydrolyze starch. Moreover, recently maltose phosphorylase was detected, which has the ability to metabolize maltose to glucose [25].

The levels of ethanol detected in the mixed-strain fermentation of ETF substrate were 44.0 and 37.5 mg/L. These levels of ethanol were within 2.7 to 65.2 mg/L, which was found in the 36-h fermentation of barley inoculated with *Lb. acidophilus* [27]. This shows that the ETF substrate has a greater ability to produce ethanol than the STF substrate using similar examined lactobacilli strains. Higher levels of lactic acid (1672 mg/L) were observed than those observed in the 36-h fermentation of barley (85 mg/L) and oats (77 mg/L) inoculated with *Lb. plantarum* [27]. Furthermore, higher lactic acid was analyzed in this study than that was reported (520 and 980 mg/L) in starch-free extract of oats inoculated with *Lb. plantarum*, and *Lb. acidophilus*, respectively. However, higher levels of acetic acid were found in oats (250 mg/L) and barley (240 mg/L) fermented with LA6. Moreover, a lower level of ethanol (0.78 mg/L) was investigated in the 10-h fermentation of the oat flour-suspension with *Lb. plantarum* [28]. In terms of flavor, organic acids are thought to contribute to the generally sour taste of lactobacillus-fermented food products [41]. It is clear that the presence of lactic acid in fermented beverages is preferable because it contributes to their mildly sour taste [40].

Lactic and acetic acids produced under the same conditions with single-strain and mixed-strain fermentations exhibited significant (*p* < 0.05) differences (Table 5, Table 8, and Table 9). The above-mentioned acids were found in greater quantities during mixed-strain fermentation. During similar fermentation, higher levels of organic acids were detected in STF than in ETF. Ethanol was produced in ETF substrates during single-strain and mixed-strain fermentations. However, this was not observed in STF. These variations might be due to the differences between accessible sugars. The ethanol detected in this study was below 0.5% *w*/*v*, which indicates that the beverage can serve as an alcohol-free alternative [9].

## 4. Conclusions

The lactobacilli strains, LA6 and LGG, which were studied, have the ability to use the available sugars in teff flours. Their growths were above 8 log cfu/mL, which is beyond the recommended minimum count of 7–6 log cfu/mL. The final cell growth observed in single-strain and mixed-strain fermentations were similar. However, the microbial growth rates, the pH drop, and the TA increase were found to be higher in mixed-strain fermentation. Consequently, they probably increase food safety along with sensory quality. A pH drop throughout the entire fermentation process would produce a harsh environment for spoilage bacteria, and it seems to be growth-limiting factor for LA6 and LGG. Maltose and glucose were not entirely consumed at the end of the exponential growth phase. Thus, the substrate concentration might not be the growth-limiting factor. The lactic and acetic acids produced during fermentation showed a hetero-fermentative metabolic pattern. The STF substrate seemed to be more suited to the growth of LA6 and LGG strains as well as to the production of metabolites. Furthermore, the findings proved that the fermentation outcome for the teff substrate inoculated with mixed strains was better than with the single strain. Finally, this research could serve as baseline information for future researchers who wish to carry out studies related to teff-based probiotic functional food products.

## Figures and Tables

**Table 1:** Completely randomized design experiments for two factors with three levels.

Experiment No.	Substrate (% *w*/*v*)	Inoculum (log cfu/mL)	Experiment No.	Substrate (% *w*/*v*)	Inoculum (log cfu/mL)
1	5.5	7	15	4	6
2	7	7	16	7	6
3	7	7	17	7	5
4	4	7	18	5.5	5
5	5.5	5	19	4	5
6	5.5	6	20	5.5	6
7	4	6	21	4	7
8	5.5	7	22	5.5	6
9	7	5	23	5.5	7
10	5.5	5	24	4	6
11	7	6	25	7	7
12	7	6	26	7	5
13	4	5	27	4	7
14	4	5			

**Table 2:** DMFit estimated kinetic growth parameters of LA6 in 24-h fermentation at 30 °C.

Inoculum (log cfu/mL)	Substrate (% *w*/*v*)	Estimated Growth Parameters		Goodness of Fit	
		Growth Rate (h^−1^)	Lag Phase (h)	R^2^	SE of Fit
5	4	0.215	3.490	0.998	0.063
	5.5	0.262	4.476	0.996	0.086
	7	0.277	4.580	0.998	0.067
6	4	0.273	4.022	0.993	0.079
	5.5	0.274	4.257	0.994	0.076
	7	0.294	4.997	0.988	0.114
7	4	0.221	3.780	0.968	0.095
	5.5	0.313	5.173	0.960	0.118
	7	0.249	3.606	0.979	0.088

Inoculum, *Lactobacillus plantarum* A6 initial density; substrate, teff flour suspension in distilled water; LA6, *Lactobacillus plantarum* A6; SE of fit, standard error of fitting.

**Table 3:** DMFit estimated kinetic parameters of pH and TA in 24-h fermentation at 30 °C.

Inoculum (Log cfu/mL)	Substrate (% *w*/*v*)	Estimated Parameters				Goodness of Fit			
		Rate (h^−1^)		Lag Phase (h)		R^2^		SE of Fit	
		pH	TA	pH	TA	pH	TA	pH	TA
5	4	−0.166	0.024	10.622	10.530	0.968	0.976	0.152	0.019
	5.5	−0.166	0.036	10.24	10.685	0.958	0.986	0.180	0.022
	7	−0.267	0.045	11.243	9.106	0.996	0.937	0.060	0.065
6	4	−0.227	0.046	4.135	5.713	0.984	0.970	0.114	0.031
	5.5	−0.218	0.033	3.997	0.000	0.987	0.972	0.104	0.045
	7	−0.244	0.040	4.464	0.000	0.990	0.964	0.094	0.063
7	4	−0.185	0.021	0.000	0.000	0.984	0.929	0.106	0.048
	5.5	−0.203	0.029	0.000	0.000	0.988	0.918	0.089	0.072
	7	−0.217	0.040	0.000	0.000	0.985	0.954	0.099	0.071

Inoculum, *Lactobacillus plantarum* A6 initial density; substrate, teff flour suspension in distilled water; TA, titratable acidity; SE of fit, standard error of fitting.

**Table 4:** Results of the Nelder–Mead simplex process and the Quality Function.

Exp.	Time (h)	Inoculum of LA6 (log cfu/mL)	Growth of LA6 (log cfu/mL)	pH	TA (g/L)	QF
1	12.000	6.000	8.078 ± 0.250 ^a,b^	4.473 ± 0.182 ^b^	0.851 ± 0.087 ^b^	1.905 ± 0.048 ^b^
2	12.000	5.500	7.781 ± 0.020 ^b^	4.840 ± 0.014 ^a^	0.622 ± 0.000 ^c^	1.799 ± 0.005 ^c^
3	13.500	6.000	8.265 ± 0.042 ^a^	4.245 ± 0.035 ^b,c^	0.901 ± 0.000 ^a,b^	1.975 ± 0.003 ^a,b^
4	13.500	6.226	8.304 ± 0.011 ^a^	4.330 ± 0.000 ^b,c^	0.901 ± 0.000 ^a,b^	1.962 ± 0.001 ^a,b^
5	15.000	6.226	8.383 ± 0.017 ^a^	4.140 ± 0.014 ^c^	1.001 ± 0.000 ^a^	2.013 ± 0.006 ^a^
6	15.000	6.000	8.400 ± 0.043 ^a^	4.2 ± 0.014 ^b,c^	1.001 ± 0.000 ^a^	2.003 ± 0.002 ^a^
7	14.250	6.068	8.346 ± 0.022 ^a^	4.335 ± 0.021 ^b,c^	1.001 ± 0.000 ^a^	1.966 ± 0.007 ^a,b^
8	14.625	6.100	8.393 ± 0.024 ^a^	4.385 ± 0.007 ^b,c^	0.975 ± 0.037 ^a,b^	1.961 ± 0.001 ^a,b^

^a–c^ All the results are the mean of two experiments ± standard deviation; LA6, *Lactobacillus plantarum* A6; TA, titratable acidity; QF, quality function; means that do not share a letter in the same column are significantly different at *p* < 0.05.

**Table 5:** Results of sugar, organic acid, and ethanol in the Nelder–Mead simplex process.

Exp.	Time (h)	Inoculum (log cfu/mL)	Maltose (mg/L)	Glucose (mg/L)	Lactic Acid (mg/L)	Acetic Acid (mg/L)	Ethanol (mg/L)
2	12	5.500	294.5 ± 3.536 ^c^	1070.5 ± 16.263 ^a^	522.0 ± 8.485 ^f^	37.5 ± 0.707 ^c^	6.0 ± 0.000 ^e^
3	13.5	6.000	318.0 ± 1.414 ^b^	557.0 ± 2.828 ^b^	820.0 ± 2.828 ^d^	43.0 ± 1.414 ^c^	19.5 ± 0.707 ^d^
4	13.5	6.226	204.5 ± 0.707 ^f^	241.0 ± 1.414 ^c^	584.0 ± 1.414 ^e^	39.0 ± 4.243 ^c^	21.5 ± 0.707 ^d^
5	15	6.226	242.0 ± 2.828 ^e^	0.0 ± 0.000 ^e^	1051.0 ± 9.899 ^b^	53.0 ± 0.000 ^b^	54.5 ± 0.707 ^a,b^
6	15	6.000	289.0 ± 5.657 ^c,d^	0.0 ± 0.000 ^e^	1140.5 ± 23.335 ^a^	82.5 ± 0.707 ^a^	48.5 ± 0.707 ^c^
7	14.25	6.068	274.5 ± 3.536 ^d^	0.0 ± 0.000 ^e^	1049.5 ± 16.263 ^b^	62.5 ± 2.121 ^b^	52.5 ± 0.707 ^b^
8	14.625	6.100	354.0 ± 7.071 ^a^	110.5 ± 2.121 ^d^	895.0 ± 14.142 ^c^	58.0 ± 4.243 ^b^	57.5 ± 2.121 ^a^

^a–f^ All the results are the mean of two experiments ± standard deviation; Inoculum, *Lactobacillus plantarum* A6 initial density; means that do not share a letter in the same column are significantly different at *p* < 0.05.

**Table 6:** Growth of LA6 and LGG in media agar at 30 °C and 37 °C under aerobiosis and anaerobiosis conditions.

Culture Media	Incubation Time (h)	LA6				LGG			
		Anaerobiosis		Aerobiosis		Anaerobiosis		Aerobiosis	
		30 °C	37 °C	30 °C	37 °C	30 °C	37 °C	30 °C	37 °C
MRS agar	24	+	+	+	+	+/−	+	−	+/−
	36	+	+	+	+	+	+	+/−	+
	48	+	+	+	+	+/−	+	+	+

MRS agar, De Man Rogosa and Sharpe agar; LA6, *Lactobacillus plantarum* A6; LGG, *Lactobacillus rhamnosus* GG; +, visible growth; +/−, weak growth; −, no visible growth.

**Table 7:** DMFit estimated kinetic parameters of growth, pH, and TA during the mixed-strain fermentation of ETF and STF substrates.

Microbes and Acidity	Estimated Parameters				Goodness of Fit			
	Rate (h^−1^)		Lag Phase (h)		R^2^		SE of Fit	
	ETF	STF	ETF	STF	ETF	STF	ETF	STF
LA6	0.350	0.326	3.044	2.49	0.999	0.995	0.034	0.078
LGG	0.357	0.482	2.860	4.04	0.994	0.998	0.079	0.050
pH	−0.484	−0.427	5.245	4.505	0.996	0.988	0.071	0.109
TA	0.070	0.112	-	4.903	0.931	0.983	0.107	0.061

LA6, *Lactobacillus plantarum* A6; LGG, *Lactobacillus rhamnosus* GG; TA, titratable acidity; ETF, teff flour samples grown in Ethiopia; STF, teff flour samples grown in South Africa; SE of fit, standard error of fitting; -, no lag phase.

**Table 8:** Results of sugar, organic acid, and ethanol in mixed-strain fermentation of ETF substrate.

Time (h)	Maltose (mg/L)	Glucose (mg/L)	Lactic Acid (mg/L)	Acetic Acid (mg/L)	Ethanol (mg/L)
0	244.5 ± 0.707 ^b^	1250.0 ± 12.728 ^c^	0.0 ± 0.000 ^d^	20.0 ± 1.414 ^d^	ND
3	244.0 ± 8.485 ^b^	1483.0 ± 33.941 ^b^	58.0 ± 4.243 ^d^	29.0 ± 1.414 ^d^	ND
6	301.5 ± 0.707 ^a^	1698.5 ± 0.707 ^a^	292.0 ± 0.000 ^c^	32.0 ± 2.828 ^c,d^	ND
9	243.5 ± 12.021 ^b^	743.5 ± 31.820 ^d^	1038.5 ± 44.548 ^b^	49.5 ± 4.950 ^b,c^	44.0 ± 2.828 ^a^
12	206.5 ± 16.263 ^c^	0.0 ± 0.000 ^e^	1180.5 ± 67.175 ^a,b^	61.5 ± 4.950 ^b^	37.5 ± 2.121 ^a^
15	124.0 ± 4.243 ^d^	0.0 ± 0.000 ^e^	1320.0 ± 48.083 ^a^	146.5 ± 9.192 ^a^	ND

^a–e^ All the results are the mean of two experiments ± standard deviation; ETF, teff flour samples grown in Ethiopia; ND, not detected; means that they do not share a letter in the same column and are significantly different at *p* < 0.05.

**Table 9:** Results obtained in the examination of sugar and organic acid in the mixed-culture fermentation of the STF substrate.

Time (h)	Maltose (mg/L)	Glucose (mg/L)	Lactic Acid (mg/L)	Acetic Acid (mg/L)
0	252.5 ± 7.778 ^a,b^	1544.0 ± 35.355 ^a^	0.0 ± 0.000 ^e^	40.0 ± 2.828 ^d^
3	232.5 ± 6.364 ^b^	1458.5 ± 45.962 ^a,b^	69.5 ± 2.121 ^e^	35.5 ± 2.121 ^d^
6	257.5 ± 0.707 ^a,b^	1435.5 ± 10.607 ^b^	373.5 ± 2.121 ^d^	52.0 ± 5.657 ^d^
9	279.5 ± 7.778 ^a^	575.5 ± 16.263 ^c^	1339.0 ± 33.941 ^b^	81.0 ± 2.828 ^c^
12	183.0 ± 0.000 ^c^	157.0 ± 0.000 ^d^	1144.5 ± 0.707 ^c^	120.0 ± 0.000 ^b^
15	236.5 ± 12.021 ^b^	0.0 ± 0.000 ^e^	1672.5 ± 36.062 ^a^	231.5 ± 12.021 ^a^

^a–e^ All the results are the mean of two experiments ± standard deviation; STF, teff flour samples grown in South Africa; means that do not share a letter in the same column and are significantly different at *p* < 0.05.

## Data Availability

Not applicable.
